# ChatGPT im Einsatz für „technology-enhanced learning“ in Anästhesiologie und Notfallmedizin und potenzielle klinische Anwendung von KI‑Sprachmodellen: Zwischen Hype und Wirklichkeit um künstliche Intelligenz im medizinischen Einsatz

**DOI:** 10.1007/s00101-024-01403-7

**Published:** 2024-05-01

**Authors:** Philipp Humbsch, Evelyn Horn, Konrad Bohm, Robert Gintrowicz

**Affiliations:** 1 Pépinière INP gGmbH, Frankfurt (Oder), Deutschland; 2 Abteilung für Anästhesiologie, Naemi-Wilke-Stift, Guben, Deutschland; 3 Klinik für Anästhesiologie m. S. operative Intensivmedizin und Prodekanat für Studium und Lehre, Charité Universitätsmedizin, Berlin, Deutschland; 4 Klinik Anästhesiologie, Intensivmedizin & perioperative Schmerztherapie, Helios Klinikum Bad Saarow, Pieskower Straße 33, 15526 Bad Saarow, Deutschland; 5 Institut für Gesundheits- und Pflegewissenschaft, Charité Berlin, Berlin, Deutschland

**Keywords:** Token, Intensivmedizin, Computerunterstütztes Lernen, Diagnostik, Lehre, Token, Intensive care, Computer-assisted learning, Diagnostics, Education

## Abstract

**Hintergrund:**

Der Einsatz von KI-Sprachmodellen in der Lehre und Wissenschaft ist aktuell Gegenstand der Forschung, und auch die Anwendung im klinischen Alltag ist in der Erprobung. Untersuchungen verschiedener Arbeitsgruppen haben gezeigt, dass Sprachmodelle Prüfungsfragen für das medizinische Staatsexamen beantworten können, und auch in der medizinischen Lehre sind Anwendungen von Sprachmodellen denkbar.

**Fragestellung:**

Es soll untersucht werden, inwiefern sich Sprachmodelle der aktuellen Version für den Einsatz bei medizinischen Fragestellungen bewähren, inwiefern sie in der medizinischen Lehre eingesetzt werden können, und welche Herausforderungen in der Arbeit mit KI-Sprachmodellen noch bestehen.

**Methode:**

Das Programm ChatGPT, basierend auf GPT 3.5, wurde genutzt, um 1025 Fragen des M2-Staatsexamens zu beantworten, und es wurde untersucht, ob und welche Fehler dabei auftraten. Außerdem wurde das Sprachmodell vor die Aufgabe gestellt, Aufsätze zu den Lernzielen der Musterweiterbildungsordnung für die Facharztweiterbildung in Anästhesiologie und die Zusatzbezeichnung in Notfallmedizin zu verfassen. Diese wurden auf Fehler und Auffälligkeiten hin untersucht.

**Ergebnis:**

Es zeigte sich, dass ChatGPT die Fragen zur mehr als 69 % richtig beantworten konnte, selbst wenn in den Aufgabenstellungen Verweise auf Abbildungen vorhanden waren. Damit konnte eine Verbesserung der Richtigkeit in der Beantwortung von Staatsexamensfragen im Vergleich zu einer Untersuchung aus dem März gefunden werden. Bei dem Verfassen von Aufsätzen zeigte sich dagegen eine hohe Fehlerrate.

**Diskussion:**

Bei dem aktuellen Tempo der fortwährenden Verbesserungen von KI-Sprachmodellen ist der breite klinische Einsatz, insbesondere in der Rettungsstelle, aber auch in der Notfall- und Intensivmedizin, bei der Arbeit von Assistenzärzten ein denkbares Szenario, die damit Hinweise für die eigene Arbeit bekommen, ohne sich nur auf das Sprachmodell verlassen zu müssen. Der Einsatz in der Lehre bedeutet für die Anwender aktuell noch einen hohen Kontrollaufwand. Aufgrund von Halluzinationen bei teils ungeeigneter Trainingsumgebung des Sprachmodells können die erstellten Texte vom aktuellen Stand der Wissenschaft abweichen. Der direkte Einsatz am Patienten außerhalb der direkten Verantwortung eines Arztes erscheint aktuell noch nicht realisierbar.

## Abstract (en)

**Background:**

The utilization of AI language models in education and academia is currently a subject of research, and applications in clinical settings are also being tested. Studies conducted by various research groups have demonstrated that language models can answer questions related to medical board examinations, and there are potential applications of these models in medical education as well.

**Research question:**

This study aims to investigate the extent to which current version language models prove effective for addressing medical inquiries, their potential utility in medical education, and the challenges that still exist in the functioning of AI language models.

**Method:**

The program ChatGPT, based on GPT 3.5, had to answer 1025 questions from the second part (M2) of the medical board examination. The study examined whether any errors and what types of errors occurred. Additionally, the language model was asked to generate essays on the learning objectives outlined in the standard curriculum for specialist training in anesthesiology and the supplementary qualification in emergency medicine. These essays were analyzed afterwards and checked for errors and anomalies.

**Results:**

The findings indicated that ChatGPT was able to correctly answer the questions with an accuracy rate exceeding 69%, even when the questions included references to visual aids. This represented an improvement in the accuracy of answering board examination questions compared to a study conducted in March; however, when it came to generating essays a high error rate was observed.

**Discussion:**

Considering the current pace of ongoing improvements in AI language models, widespread clinical implementation, especially in emergency departments as well as emergency and intensive care medicine with the assistance of medical trainees, is a plausible scenario. These models can provide insights to support medical professionals in their work, without relying solely on the language model. Although the use of these models in education holds promise, it currently requires a significant amount of supervision. Due to hallucinations caused by inadequate training environments for the language model, the generated texts might deviate from the current state of scientific knowledge. Direct deployment in patient care settings without permanent physician supervision does not yet appear to be achievable at present.

## Einleitung

Die Maschine als Kommunikator, Computerprogramme für die Erzeugung von Bild, Ton und Texten – kurz künstliche Intelligenz (KI) – ist gerade dabei, eine neue digitale Revolution voranzutreiben, die Maschinen befähigt, eigene Inhalte zu erstellen, mit Menschen in natürlich wirkender Weise zu kommunizieren und gewaltige Mengen an Daten zu analysieren und zu verarbeiten. Viele dieser Technologien sind, wie ChatGPT, im Internet frei zugänglich und haben die Nutzung dieser Möglichkeiten der breiten Öffentlichkeit zugänglich gemacht.

## Die Technologie hinter den Sprachmodellen

**Figure 1:**
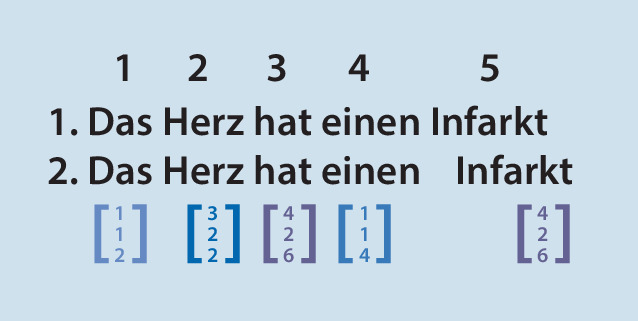

**Figure 2:**
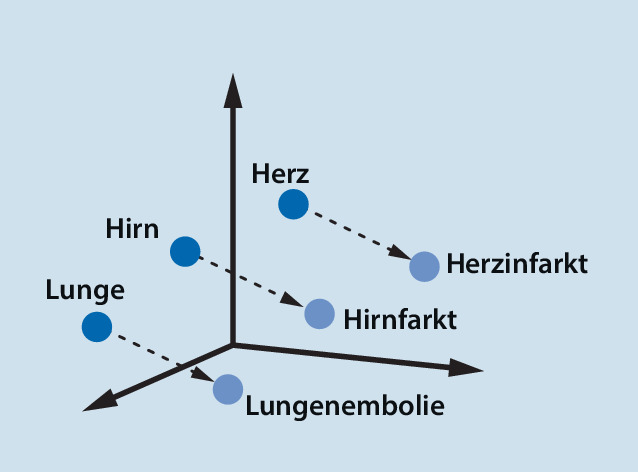

Künstliche Intelligenz kann je nach Form der zu verarbeitenden Information verschiedene Wege in der Prozessierung nehmen. ChatGPT beruht aktuell in der kostenlosen Version auf dem GPT‑3.5‑Sprachmodell (Generative Pre-trained Transformer 3.5); das ist ein autoregressives Sprachmodell, dass natürlich wirkende Texte erzeugen kann. Bei autoregressiven Modellen basiert der ausgegebene Wert zu einem Zeitpunkt t_0_ auf einer Linearkombination von vorhergehenden gegebenen Werten (Vorwärtsprädiktion) oder auf einer Linearkombination nachfolgender Werte (Rückwärtsprädiktion). Ebenso ist eine Kombination beider (Vorwärts-Rückwärts-Prädiktion) möglich. Der gegebene Text wird in Wörter und Zeichen zerlegt; diese Bruchstücke des Textes werden Token genannt. Den Token werden dabei verschiedene Kategorien zugeordnet, und anhand ihrer Position im Satz die wahrscheinlichsten Wörter vor und nach dem Wort bestimmt. Um die Wörter dabei aber im Kontext zu erfassen, müssen ihnen für das maschinelle Lernen Vektoren zugeordnet werden (Abb. 1), Synonyme haben dabei immer gleiche Vektoren. Damit erfassen die Sprachmodelle nicht nur das gegebene Wort, sondern auch die Wörter vor und hinter dem Wort und stellen diese in Beziehung zueinander. Dass der Kontext insbesondere in der deutschen Sprache wichtig ist, lässt sich an einem Beispiel erklären: Der Fall eines Patienten kann den physischen Sturz aus einer gewissen Höhe meinen oder die andere Bedeutung haben, dass damit der gesamte Vorgang seiner Behandlung innerhalb einer Einrichtung gemeint ist. Das Sprachmodell kann also Inhalte anhand ihrer semantischen Aussagen zueinander kategorisieren und so auch erkennen, ob sie zueinander gleich, neutral oder gegensätzlich sind. Mithilfe dieser Token kann das Sprachmodell nun das wahrscheinlichste nächste Wort im Kontext aller Token berechnen. Um zu wissen, welche Wörter und Zeichen hierbei in Beziehung stehen, müssen Sprachmodelle trainiert werden. Dabei werden Petabytes an Texten auf ihre statistische Verteilung der einzelnen Token untersucht und dann diese Token miteinander in Beziehung gesetzt (Abb. 2 und 3). Am besten nachvollziehen lässt sich diese Vorhersage von passenden Wörtern durch natürliche Sprachverarbeitung („natural language processing“, NLP) bei der Autokorrektur in Suchmaschinen und bei Textnachrichtenprogrammen, die trotz Schreibfehler das richtige Wort anbieten und sogar teilweise das nächste Wort vorschlagen. Diese Analyse der Wörter eines gegebenen Textes durch immer weiter aufteilendes Kategorisieren in Token, die aus Wörtern, Silben und Zeichen bestehen, wird als tiefes Lernen („deep learning“) bezeichnet, weil die Information in immer neue Schichten aufgeteilt wird.

**Figure 3:**
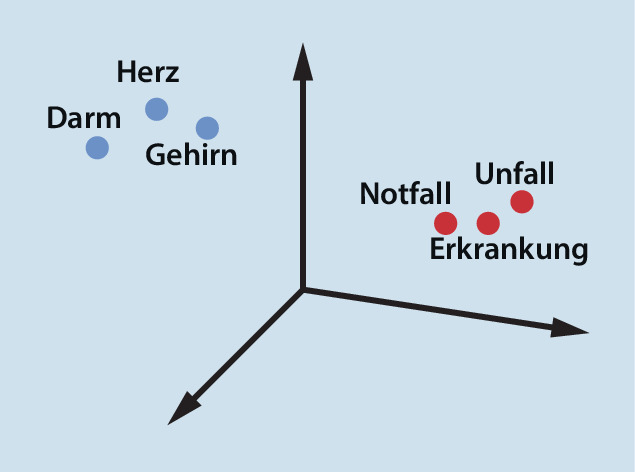

## KI im Einsatz in Klinik und Lehre

Die KI-gestützte Auswertung digitaler Daten in der Medizin ist ein wichtiges Forschungsgebiet [1–3] und ein stetig wachsender Markt für junge Start-ups. Ob bei der Vorhersage des Todeszeitpunktes [4], in der Dermatologie [5, 6], beim Management der Schlafapnoe [7], dem Schreiben von Arztbriefen [8] oder als Werkzeug in der Radiologie [9, 10], aktuell werden viele Anwendungsmöglichkeiten für den Einsatz von KI im klinischen Bereich untersucht und erprobt. Bei der Erstellung von Texten für Abschlussarbeiten bzw. der Kontrolle dieser Arbeiten auf durch KI erzeugte (Plagiat‑)Texte kommt wiederum KI zum Einsatz [11, 12]. Auch beim Erstellen von Erklärvideos aus Texten wird KI bereits zum Einsatz gebracht [13]. Ebenso kamen Sprachmodelle bereits bei der experimentellen Bearbeitung von Prüfungsfragen [14, 15], bei denen vorher alle Bildfragen ausgeschlossen wurden, zum Einsatz. Hierbei wurde gezeigt, dass ChatGPT diese Fragen in den meisten Fällen richtig beantworten kann. Anhand dieser Untersuchung beschrieben die Autoren Jung et al. [15] „die Fähigkeit von LLMs (großen Sprachmodellen), medizinische Daten zu strukturieren und Informationen vor dem Hintergrund der verfügbaren Literatur zu interpretieren“. Diese würde das Potenzial für die Nutzung von ChatGPT in der Medizin bergen. Sie regten außerdem an, dass „künftige Arbeiten (…) die Leistung von KI-Anwendungen bei Bildfragen sowie unterschiedlichen Fragetypen untersuchen“ sollten. Außerdem wird durch eine größere Stichprobe die Fähigkeit zur reflektierten Beantwortung der Fragen untersucht. Ferner wurde untersucht, inwiefern solche Sprachmodelle für die Erstellung von Texten für Abschlussarbeiten und in der Lehre geeignet sind, und inwiefern die Antworten von Chat GPT für die Beantwortung von medizinischen Fragen im klinischen Einsatz geeignet sein können.

## Material und Methode

Die Analysen zu den Prüfungsfragen beruhen auf den bei Amboss (www.amboss.com, AMBOSS GmbH, 19.08.2023) zugänglichen Prüfungsfragen zum M2-Staatsexamen. In manchen Fällen beinhalten die Fragen dabei Abbildungen und Bilder, die für die Beantwortung der Frage notwendig oder hilfreich sein können. ChatGPT bietet keine Möglichkeit, Abbildungen in Fragen einzufügen, gleichwohl könnte hier aber ein möglicher Indikator bestehen, um die selbstreflektierte Beantwortung von Fragen zu kontrollieren: Fragen (*n* = 120), in denen die Abbildungen zwar enthalten, aber nicht zwingend zur Beantwortung benötigt werden, und Fragen (*n* = 46), in denen die Abbildungen für die Beantwortung notwendig sind, werden in die Untersuchung miteinbezogen und gesondert erfasst. Jede Frage wurde einem Fachgebiet zugeordnet. Außerdem wurde zwischen Fragen mit Quellenbezug (*n* = 450, z. B. Fallvignette oder Abbildung) und Fragen ohne Quellenbezug unterschieden. Die Untersuchung wurde mit ChatGPT, basierend auf GPT 3.5 (https://chat.openai.com/; Version: 03.08.2023; OpenAI), durchgeführt. Es wurden 1025 Fragen untersucht. Die Fragen wurden eingegeben und mit den Lösungen des Examens auf der Amboss-Plattform verglichen. Die Ergebnisse wurden mithilfe des Chi-Quadrat-Tests mit denen der Arbeit von Jung et al. verglichen. Die Fälle, in denen ChatGPT eine Frage, die es wegen der fehlenden Abbildung nicht richtig beantworten konnte, ablehnte oder trotzdem beantwortete, wurden erfasst. Manchmal gab ChatGPT nicht nur die Antwort, sondern auch einen Begründungstext aus. Bei falschen Antworten wurde dieser ebenfalls auf das Vorliegen von Halluzinationen untersucht. Als Halluzinationen werden erfundene Inhalte bezeichnet, die durch die KI erzeugt wurden, aber in der Realität keine Entsprechung finden.

Für die Untersuchung zur Erstellung von Lerninhalten wurde auf die Spalte „Kognitive und Methodenkompetenz – Kenntnisse“ der Musterweiterbildungsordnung der Bundesärztekammer von 2018 für die Facharztweiterbildung Anästhesiologie [11] und die Zusatzbezeichnung Notfallmedizin [18] zurückgegriffen (Tab. 1). Die Aufsätze wurden von ChatGPT ausgegeben und mittels einer 5‑gliedrigen Likert-Skala unter dem Aspekt „Richtigkeit der dargestellten Fakten“ bewertet. Hierfür wurden die Texte von 3 Untersuchern (Ärzte mit wenigstens 2 Jahren Berufserfahrung) diskutiert; die Bewertung erfolgte einstimmig. Die so ermittelten Attribute wurden dann mittels Microsoft Excel® (Version 2019, Microsoft Corporation, Redmond, USA) auf ihren Median untersucht.

**Table 1:** 

	Kognitive und Methodenkompetenz – Kenntnisse aus der Musterweiterbildungsordnung der Bundesärztekammer von 2018							
	Anästhesiologie				Notfallmedizin			
	Lernzielkatalog	Ergebnis*	Feinlernziel	Ergebnis*	Lernzielkatalog	Ergebnis*	Feinlernziel	Ergebnis*
1	Wesentliche Gesetze, Verordnungen und Richtlinien	1	Fachaufsatz zum Thema ärztliche Delegation bei der Narkose	4	Wesentliche Gesetze, Verordnungen und Richtlinien, z. B. Rettungsdienstgesetze	1	Fachaufsatz über die Unterschiede in den Qualifikationen der Rettungsdienstmitarbeiter nach Rettungsassistentengesetz oder Notfallsanitätergesetz	3
2	Anästhesierelevante Ultraschallverfahren, insbesondere Notfallsonographie, transösophageale und transthorakale Echokardiographie	1	Fachaufsatz zum Thema FAST-Sonographie	5	Strukturen des deutschen Rettungsdienstes sowie Indikationen der verschiedenen Rettungsmittel	1	Fachaufsatz zu den gültigen Indikationen für den Notarzteinsatz im Rettungsdienst durch die Leitstelle	3
3	Risiken und Vorteile unterschiedlicher anästhesiologischer Verfahren bei neurochirurgischen und neurointerventionellen Eingriffen	1	Fachaufsatz zum Thema Beatmungsprobleme bei Eingriffen an der Halswirbelsäule	2,5	Einsatzarten, insbesondere Primär‑, Sekundäreinsatz, Interhospital- und Schwerlasttransport, Infektionstransport, Neugeborenentransport	1	Fachaufsatz über die Unterschiede und Gemeinsamkeiten eines Primär- und Sekundäreinsatzes im Rettungsdienst	4,5
4	Prinzipien und Besonderheiten der Anästhesiologie bei intrakraniellen Eingriffen	3	Fachaufsatz zum Thema Senkung des Hirndrucks in der Anästhesiologie	5	Aufgaben und Struktur einer Leitstelle, der Alarmierungswege und Alarmierungsmittel	1	Fachaufsatz über die Alarmierungswege einer Leitstelle bei einem Massenanfall von Verletzten (MANV)	3
5	Besonderheiten der pädiatrischen Anästhesiologie, einschließlich Monitoring, Atemwegsmanagement, i.v.- und i.o.-Zugänge, Narkoseeinleitung, -aufrechterhaltung, -ausleitung, postanästhesiologische Versorgung, Flüssigkeits- und Volumentherapie	3	Fachaufsatz über die Dosierung der narkoserelevanten Medikamente bei Einleitung und Aufrechterhaltung der Narkose bei Patienten im Kindesalter bis 5 Jahren	2	Besonderheiten und Kontraindikationen bei ambulanter notärztlicher Versorgung	2	Fachaufsatz über die notwendigen Voraussetzungen für ein Belassen des Patienten in seiner Häuslichkeit durch den Notarzt	2
6	Prinzipien und Besonderheiten der Anästhesiologie bei thoraxchirurgischen Eingriffen	3	Fachaufsatz zum Thema Besonderheiten einer Intubation mit dem Doppellumentubus	5	Möglichkeiten einer ambulanten Weiterversorgung durch Hausarzt, sozialpsychiatrischen Dienst, spezialisierte ambulante Palliativversorgung oder Sozialstation	2	Fachaufsatz über die Möglichkeiten der palliativmedizinischen Anbindung bei Notfallpatienten mit lebenszeitverkürzenden Krebserkrankungen im Endstadium	3,5
7	Perioperative Schmerztherapie, einschließlich epiduraler, para- und intervertebraler Blockaden in der Thoraxchirurgie	3	Fachaufsatz zur Anlage einer periduralen Anästhesie bei einseitigen Lobektomien	3	Grundlagen der technischen und medizinischen Rettung	3	Fachaufsatz über die Kontraindikationen für einen Patiententransport im Rettungshubschrauber	1
8	Prinzipien und Besonderheiten der Anästhesiologie bei kardiochirurgischen und herznahen gefäßchirurgischen Eingriffen, insbesondere des kardiopulmonalen Bypass und anderer kreislaufunterstützender Maßnahmen	3	Fachaufsatz zu den speziellen anästhesiologischen Risiken bei Klappenoperationen am offenen Herzen	3,5	Grundlagen der Lagebeurteilung und Sichtung bei einem Massenanfall von Verletzten/Erkrankten (MANV), auch unter chemischen/biologischen/radiologischen/nuklearen (CBRN)-Gefahren	3	Fachaufsatz über die Vorgehensweise für den ersteintreffenden Notarzt bei einem MANV durch einen großen Verkehrsunfall auf einer Autobahn	4
9	Mindestanforderungen für die Anwendung anästhesiologischer Verfahren bei ambulanten Eingriffen	3	Fachaufsatz über die anästhesiologischen Ausschlusskriterien von ambulanten Eingriffen an Gelenken	3	Grundlagen des Katastrophenschutzes	2	Fachaufsatz zu den Einsatzmöglichkeiten einer schnellen Einsatzgruppe (SEG) im Katastrophenschutz	3
10	Perkutane Tracheotomien	3	Fachaufsatz über die praktische Durchführung einer perkutanen Tracheotomie in der intensivmedizinischen Versorgung	1,5	Auswahl eines dem Krankheitsbild entsprechend leitliniengerechten und geeigneten Zielkrankenhauses	1	Fachaufsatz über die Anforderung an ein Zielkrankenhaus für Patienten mit Schussverletzungen	3
11	Grundlagen der Behandlung chronischer Schmerzen	2	Fachaufsatz zur medikamentösen Therapie von chronischen Schmerzen bei Patienten mit Gelenkbeteiligung	3,5	Bedeutung notfallmedizinisch relevanter Register (Reanimations‑, Traumaregister) und Dokumentationsgrundlagen (MIND)	2	Fachaufsatz über den Nutzen des Reanimationsregisters für die Arbeit im Rettungsdienst	1,5
12	–	–	–	–	Bedeutung und Indikation von Krisenintervention und Einsatznachsorge	3	Fachaufsatz über die Möglichkeiten der Einsatznachsorge bei belastenden Einsatzgeschehen	4
13	–	–	–	–	Situation des rechtfertigenden Notstandes und der Geschäftsführung ohne Auftrag	2	Fachaufsatz über die notwendigen Bedingungen für den rechtfertigenden Notstand bei der Behandlung von Patienten	4,5
14	–	–	–	–	Besonderheiten bei der Unterbringung psychisch Kranker nach gesetzlichen Regelungen	3	Fachaufsatz zu den notwendigen Bedingungen für eine Anwendung des PsychKG	4,5
15	–	–	–	–	Schockraummanagement	3	Fachaufsatz über die wichtigsten Merkmale der Übergabesituation vom Notarzt an den Schockraumleiter	4
16	–	–	–	–	Grundlagen der transkutanen Schrittmachertherapie	1	Fachaufsatz über die Vorgehensweise der transkutanen Schrittmachertherapie bei kreislaufwirksamer bradykarder Herzrhythmusstörung	2
17	–	–	–	–	Besonderheiten und Ablauf einer Neugeborenenerstversorgung	1	Fachaufsatz über die Wahl des Zugangs zum Kreislaufsystems bei einer Neugeborenenreanimation	5
18	–	–	–	–	Geburtshilfliches Notfallmanagement	2	Fachaufsatz über die Geburtsunterstützung durch den Notarzt bei einer Geburt mit vorderer Hinterhauptslage	1,5

Außerdem wurde ChatGPT aufgefordert, Texte zu formulieren zu Feinlernzielen aller Lerninhalte der Musterweiterbildungsordnung, die auf diesen Lernzielen beruhen, aber in der Fragestellung tiefer gehen. Auch diese wurden wie die anderen Texte durch dieselben Untersucher bewertet (Tab. 1). Diese Feinlernziele richteten sich nach den Lernzielen des Kataloges, wurden aber in Erweiterung dessen zu einem hierzu passenden Thema gefordert. Sie wurden durch dieselben Ärzte einstimmig bewertet. Die Ärzte haben bereits die Zusatzweiterbildung Notfallmedizin abgeschlossen, oder sind weit fortgeschritten in ihrer Ausbildung zum Facharzt in Anästhesiologie. Die Ergebnisse beider Fragetypen wurden innerhalb der Fachgebiete mit dem Chi-Quadrat-Test auf das Vorliegen eines signifikanten Unterschieds hin untersucht.

## Ergebnisse

Insgesamt wurden 1025 Fragen aus 29 Fachgebieten gestellt, davon bezogen sich 450 Fragen auf Quellen. Es wurden 69,5 % aller Fragen richtig beantwortet. Bei Fragen mit Quellenverweisen wurden 289 (64,2 %) richtig beantwortet, 140 Fragen (31,1 %) wurden falsch beantwortet, und bei 21 Fragen (4,7 %) wurde eine Beantwortung mit Verweis auf die fehlende Quelle abgelehnt. Bei den 140 Fragen konnten 126 (90 %) auch ohne Quelle allein anhand des Fragentextes richtig beantwortet werden, während bei 14 (10 %) der falsch beantworteten Fragen und 20 (95,2 %) der abgelehnten Fragen die Quelle für die Beantwortung der Frage zwingend notwendig war.

Bei 252 (86,6 %) aller falsch beantworteten Fragen wurde lediglich die falsche Antwort ausgegeben, bei 39 (13,4 %) wurde zusätzliche eine in Teilen oder komplett falsche oder widersprüchliche Begründung zur Antwort dazu ausgegeben. Hierbei wurde 15-mal eine falsche Diagnose begründet, 8‑mal wurden falsche physiologische Angaben gemacht, 5‑mal eine falsche Beratung zum ärztlichen Vorgehen gegeben und 10-mal eine falsche Therapie begründet. Während 35 (89,7 %) der Fragen lediglich falsche Begründungen angaben, wurden bei 4 Fragen (10,3 %) Sachverhalte oder Fakten halluziniert.

**ChatGPT besteht in Notfall- und Intensivmedizin, fällt aber in Anästhesiologie und Rechtsmedizin durch** **.**

In 8 Fachgebieten wurden mehr als 60 Fragen gestellt; Spitzenreiter war dabei die Innere Medizin mit 172 Fragen, gefolgt von Notfallmedizin (106), Genetik (94), Intensivmedizin (87), Neurologie (83), Anästhesiologie (69), Psychologie/Psychiatrie (67) und Rechtsmedizin (61) (Tab. 2).

**Table 2:** 

Fachgebiete	Richtig	%	FALSCH	%	Abgelehnt	%	Gesamt	%
Innere Medizin	131	76,2	38	22,1	3	1,7	172	100
Notfallmedizin	79	74,5	27	25,5	0	0,0	106	100
Genetik	59	62,8	34	36,2	1	1,1	94	100
Intensivmedizin	67	77,0	19	21,8	1	1,2	87	100
Neurologie	58	69,9	21	25,3	4	4,8	83	100
Anästhesiologie	39	56,5	29	42,0	1	1,5	69	100
Psychologie	54	80,6	13	19,4	–	0,0	67	100
Rechtsmedizin	25	41,0	36	59,0	0	0,0	61	100
Ärztliches Handeln	39	76,5	12	23,5	–	0,0	51	100
Pharmakologie	21	77,8	4	14,8	2	7,4	27	100
Radiologie	10	47,6	8	38,1	3	14,3	21	100
Epidemiologie	19	90,5	2	9,5	–	0,0	21	100
Unfallchirurgie/Orthopädie	10	47,6	10	47,6	1	4,8	21	100
Chirurgie	16	80,0	3	15,0	1	5,0	20	100
Pädiatrie	13	65,0	7	35,0	–	0,0	20	100
Medizinrecht	16	84,2	3	15,8	0	0,0	19	100
Gynäkologie	11	61,1	7	38,9	0	0,0	18	100
Dermatologie	9	64,3	3	21,4	2	14,3	14	100
Arbeitsmedizin	7	77,8	2	22,2	–	0,0	9	100
Augenheilkunde	5	55,6	3	33,3	1	11,1	9	100
Pathologie	7	87,5	0	0,0	1	12,5	8	100
Urologie	6	85,7	1	14,3	0	0,0	7	100
Neurochirurgie	5	83,3	0	0,0	1	16,7	6	100
Neonatologie	2	40,0	3	60,0	–	0,0	5	100
Anatomie	0	0,0	4	100,0	–	0,0	4	100
HNO	2	66,7	1	33,3	–	0,0	3	100
Nuklearmedizin	1	100,0	0	0,0	0	0,0	1	100
Strahlentherapie	0	0,0	1	100,0	–	0,0	1	100
Naturheilkunde	1	100,0	0	0,0	0	0,0	1	100
Gesamtergebnis	712	69,5	291	28,4	22	2,1	1025	100,0

Diese 8 Fachgebiete brachten es in der Untersuchung auf 739 Fragen; von denen wurden 67,3 % richtig und 31,4 % falsch beantwortet. Beim Rest (1,3 %) wurde eine Beantwortung abgelehnt.

Am meisten richtige Antworten gab es in der Psychologie/Psychiatrie (80,6 %), gefolgt von Intensivmedizin (77 %), Innerer Medizin (76 %), Notfallmedizin (74,5 %), Neurologie (69,9 %), Genetik (62,8 %), Anästhesiologie (56,5 %) und Rechtsmedizin (41 %).

## Qualitative Auswertung der Aufsätze

**Figure 4:**
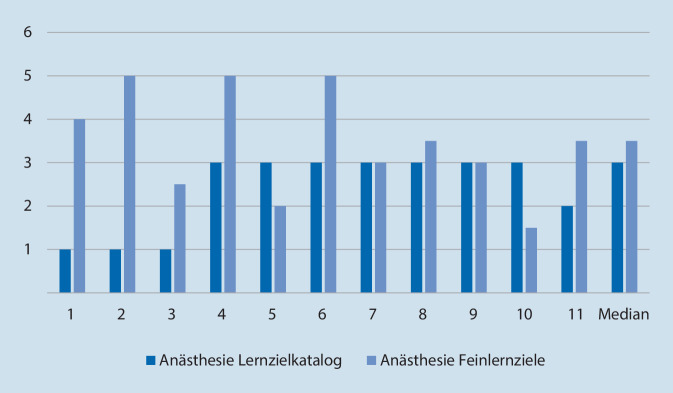

Für die zwei Bereiche Anästhesiologie und Notfallmedizin wurden insgesamt 59 Aufsatzanfragen an ChatGPT gestellt (jeweils 18 für die Notfallmedizin und 11 für die Anästhesiologie zu den Lernzielen des Lernzielkataloges der Weiterbildungsordnung sowie zu den daraus abgeleiteten Feinlernzielen). Diese Aufsätze wurden nach dem Aspekt „Richtigkeit“ mit ganzzahligen Werten von 1 bis 5 durch alle Untersucher bewertet, daraus wurde dann der Durchschnitt errechnet. Der Punktwert 5 war der höchste zu erzielende und folglich 1 der niedrigste Punktwert. Für die Richtigkeit wurde bei der Anästhesiologie der Median von 3 erzielt. Im Fach Notfallmedizin lag bei der Richtigkeit der Median bei 2 (Abb. 4 und 5). Bei den Aufsätzen zu den Feinlernzielen, die sich von den Lernzielen der Weiterbildungsordnung ableiten, lag der Median in der Anästhesiologie bei 3,5 und in der Notfallmedizin bei 3. Für die Ergebnisse der Anästhesiologie lag *p* bei 0,384, bei der Notfallmedizin bei 0,29; die gefundenen Unterschiede waren insofern nicht signifikant.

**Figure 5:**
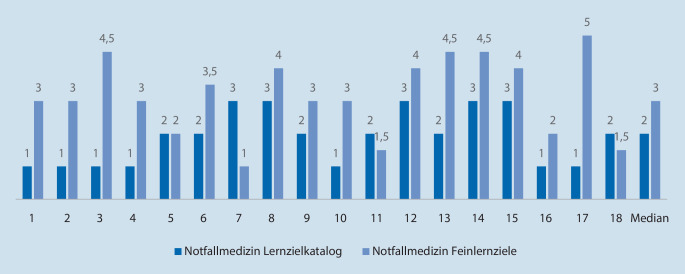

## Diskussion

Die Bestehensgrenzen für die Examina wurden durch das IMPP mit 60 % [16] angegeben, und es zeigte sich, dass ChatGPT diese Schwelle bei den 1025 gestellten Fragen erfolgreich überschreiten konnte, selbst wenn die Fragen mit Bilderverweis nicht ausgeschlossen wurden. Dass ChatGPT diese Grenze erreichen würde, war nach den Untersuchungen von Kung et al. [14] und Jung et al. [15] zu erwarten. Es zeigten sich Unterschiede in den Ergebnissen nach Fachgebieten (Tab. 3). Wie viele Fragen dabei mit denen von Jung et al. übereinstimmten, ist jedoch nicht zu ermitteln.

**Table 3:** 

	Fragen bei Jung et al. [15]		Fragen in der aktuellen Untersuchung		
Fachgebiete	Absolute Zahl der Fragen	Anteil richtiger Antworten	Absolute Zahl der Fragen	Anteil richtiger Antworten	*p*-Werte
Augenheilkunde	7	85,7	5	55,6	< 0,01
AINS	8	75	185	71,6	< 0,01
Chirurgie/Orthopädie	14	64,3	26	67,5	0,02
Dermatologie	7	85,7	9	64,3	< 0,01
Epidemiologie	15	46,7	19	90,5	< 0,01
Gynäkologie	20	80	11	61,1	< 0,01
HNO	3	33,3	3	66,7	< 0,01
Humangenetik	14	64,3	59	62,8	< 0,01
Innere Medizin und Infektiologie	47	70,2	131	76,2	< 0,01
Neurologie	46	46,7	58	69,9	< 0,01
Pädiatrie	23	65,2	13	65	0,01
Pharmakologie	19	94,7	21	77,8	< 0,01
Psychiatrie	9	66,7	54	80,6	< 0,01
Radiologie	8	75	10	47,6	< 0,01
Rechtsmedizin	12	66,7	25	41	< 0,01
Urologie	1	100	6	85,7	0,01

**Figure 6:**
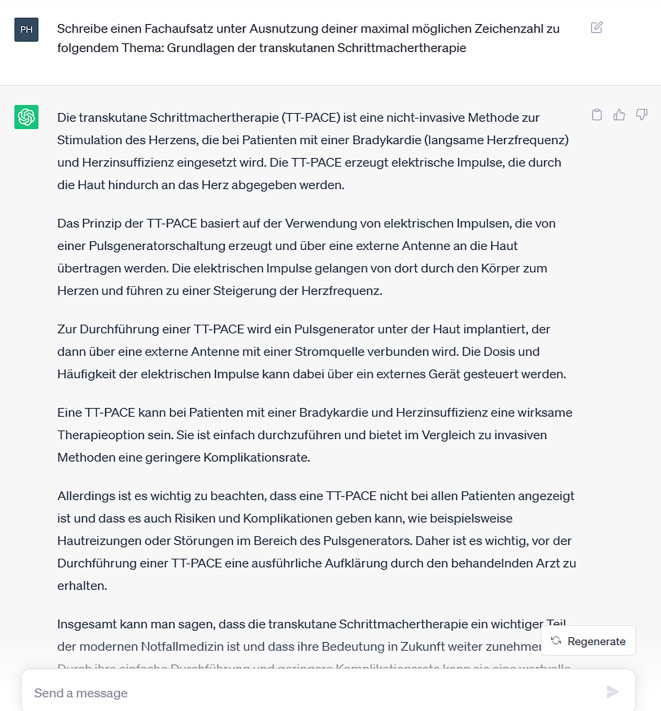

Im Gegensatz zu Jung et al. konnte die KI den Anteil richtiger Fragen sogar noch erhöhen, und wenn man alle Fragen ausschließen würde, die ohne eine Abbildung nicht zu beantworten waren, steigt der Anteil richtiger Antworten sogar auf 71,1 % gegenüber 66,7 % bei Jung et al. (*p* < 0,01). Das zeigt, dass die Leistungsfähigkeit des Sprachmodells bereits in der kurzen Zeit seit März 2023 signifikant zugenommen hat, was an den fortwährenden Verbesserungen der Software (Updates) liegen kann. Die stetige Zunahme der Leistungsfähigkeit der zugrunde liegenden Sprachmodelle ist für die Anwendung in der Klinik und Lehre von herausragender Bedeutung und macht bereits jetzt einige Anwendung möglich, auch wenn es in erster Linie um Bildverarbeitung geht, wie etwa in der Radiologie und nicht um Texte wie bei ChatGPT. Für Anwendungen in der Lehre bedarf es nach aktuellem Stand noch der menschlichen Supervision. Es zeigte sich, dass die von ChatGPT erstellten Inhalte in mancher Hinsicht vom Stand der Wissenschaft abwichen. Besonders eindrücklich ist hierbei der Aufsatz zur transkutanen Schrittmachertherapie (Abb. 6). Hier empfahl ChatGPT das transthorakale Pacing (TT-PACE). Die Anwendung des TT-PACE erklärte ChatGPT wie folgt: „Zur Durchführung einer TT-PACE wird ein Pulsgenerator unter die Haut implantiert, der dann über eine externe Antenne mit einer Stromquelle verbunden wird.“ Dies solle den Nervus vagus stimulieren, so das Sprachmodell, um u. a. Herzrhythmusstörungen zu therapieren. Den Widerspruch zwischen der Implantation unter die Haut und einer transkutanen Stimulation artikulierte ChatGPT nicht. Bei der Recherche auf PubMed zeigten sich zum „TT-PACE“ keine Einträge, zur Nervus-vagus-Stimulation (VNS) einige Publikationen, sogar zum Einsatz bei kardiovaskulären Erkrankungen [17] – aber eben nicht zur Schrittmachertherapie. Einige Monate später gab eine neuere Version des Sprachmodells richtigerweise an, dass TT-PACE kein standardmedizinischer Begriff sei, ließ sich dann aber durch eine Suggestivfrage wieder zur Reproduktion der alten, falschen Antwort verleiten.

**Figure 7:**
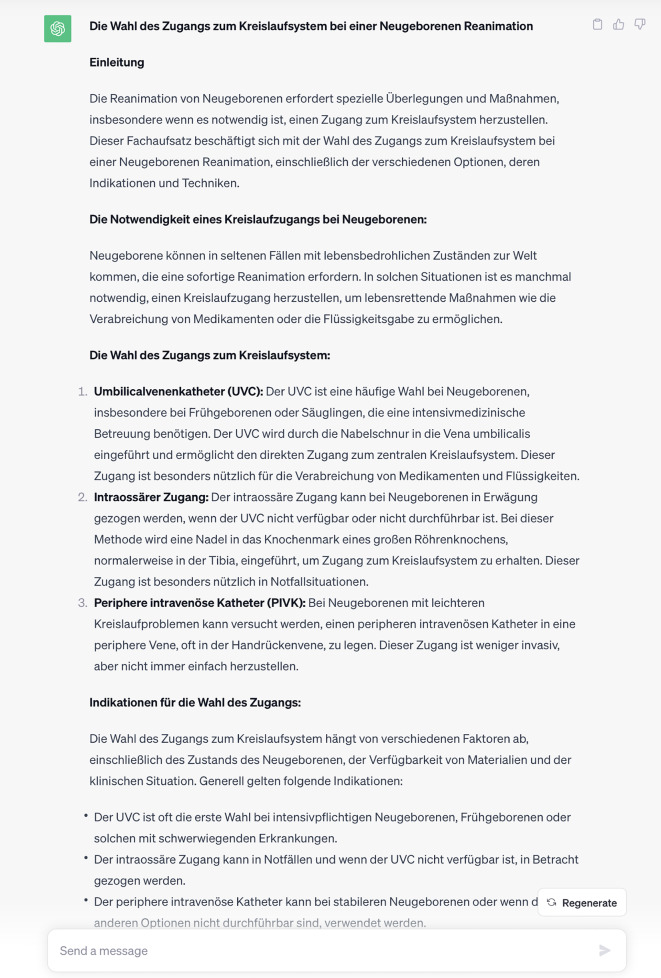

An anderer Stelle beschreibt ChatGPT den Transport von Notfallpatienten im Notarzteinsatzfahrzeug: „So werden beispielsweise schwerwiegende Verletzungen oder akute Erkrankungen, bei denen eine schnelle medizinische Versorgung notwendig ist, häufig mit dem Notarzteinsatzfahrzeug oder dem Rettungshubschrauber transportiert.“ Dass das Notarzteinsatzfahrzeug nicht für den Patiententransport gedacht ist, sondern dem Transport des Notarztes zum Patienten dient, gab ChatGPT nicht richtig wieder. Bei den Aufsätzen zu den Feinlernzielen konnte hierzu keine signifikante Verbesserung gefunden werden. Zwar liegt die Vermutung nahe, dass das Sprachmodell durch die konkretere Fragestellung mit mehr Informationen eine bessere Antwort generieren kann, das ist jedoch bei dem gewählten Versuchsaufbau nicht nachweisbar. Ein Grund hierfür kann die englischsprachige Lernumgebung des Sprachmodells sein, wobei die deutschen Fragen in englische Sprache übersetzt werden, hier kann es zu Unschärfen kommen, die wiederum ihrerseits zu Fehlern bei der Antwort führen können. Während die Software bei manchen Fragen den aktuellen Stand von Leitlinien weiterzugeben wusste, erklärte sie in einem Aufsatz zu einem Feinlernziel, beruhend auf dem Lernziel „geburtshilfliches Notfallmanagement“: „Eine vordere Hinterhauptslage tritt auf, wenn sich das Baby mit dem Hinterkopf nach unten, aber mit dem Gesicht nach vorne in Richtung des mütterlichen Rückens befindet. Dies ist eine ungewöhnliche Position, da normalerweise der Hinterkopf nach unten und das Gesicht zur Wirbelsäule des Babys zeigt“, der semantische Fehler in diesen Zeilen wurde durch die Software nicht zuverlässig erkannt. An anderer Stelle arbeitet die Software hingegen einen sehr schlüssigen und dem Stand der Wissenschaft entsprechenden Text aus, wie etwa bei der Wahl des Zugangs zum Gefäßsystem bei der Reanimation von Neugeborenen (Abb. 7). Dies könnte an der Menge an Quellen liegen, die hierzu in der Trainingsumgebung den richtigen Sachverhalt wiedergegeben haben. Wenn der Anteil geeigneter Quellen in der Trainingsumgebung hoch genug sein sollte, wäre es hinreichend wahrscheinlich, dass die Software entsprechende Token produziert, die in entsprechender semantischer Beziehung zueinander stehen. Welche Faktoren aber letztlich beeinflussen, wann die Sprachmodelle zu einem schlüssigen, inhaltlich korrekten Text in der Lage sind und wann nicht, ist weiterhin unklar und bedarf weiterer Untersuchungen hierzu.

Die Software steht dabei vor unterschiedlichen Herausforderungen. Zusätzlich zur überwiegend englischsprachigen Lernumgebung (auch wenn Open AI hier die genauen Anteile nicht veröffentlicht) wird die maximale Zeichenlänge je Antwort auf 2048 Zeichen begrenzt. Zwar kann das Sprachmodell auch längere Antworten ausgeben, in dem es diese auf mehrere Antworten aufteilt, jedoch kommt es hierbei nicht selten zu Syntaxfehlern, die den Lesefluss stören oder den ganzen Text zusammengenommen widersprüchlich erscheinen lassen. Auch ist nicht auszuschließen, dass jede gestellte Frage die nachfolgenden Fragen beeinflusst, unabhängig davon, ob für jede Frage ein neuer Chat geöffnet wird oder nicht, da die KI alle Fragen und Antworten abspeichert und die dabei erzeugten Token wiederum für Herstellung von Beziehungen heranziehen kann (Beeinflussung der Lern- und Trainingsumgebung durch die Fragen der Anwender).

Aber auch unabhängig von der Lern- und Trainingsumgebung kann ChatGPT Fehler (Halluzinationen) produzieren, und diese Halluzinationen sind weder vorhersagbar noch immer begründbar.

**Autoregressive Modelle könnten anfälliger bei Aufgaben mit wenigen Begleitinformationen sein.**

Die Fehleranfälligkeit war bei den Aufsätzen zu den Lernzielen (sowohl denen aus der Weiterbildungsordnung als auch zu den daraus abgeleiteten Feinlernzielen) bedeutend höher als bei den Aufsätzen zu den klinischen Fragen des Staatsexamens. Ein Grund kann hierbei in der zugrunde liegenden Autoregression liegen. Je mehr Token aus dem eingegebenen Text erzeugt werden können, desto mehr semantische Beziehungen kann das Sprachmodell für die Erzeugung des Textes nutzen. Sollte sich diese Vermutung bestätigen, wäre die Anwendung von Sprachmodellen im klinischen Rahmen dem generellen Einsatz in der Lehre schon deshalb überlegen, weil in aller Regel zu einem Patienten mehr Befunde erhoben werden (können), als bei Fragestellungen zu Lerninhalten eingebracht werden. Zwar ließe sich das durch ausreichende Begleitinformationen zur Fragestellung kompensieren, jedoch scheint der Aufwand zur Kontrolle der erstellten Texte aufgrund ihrer Länge größer im Vergleich zur Kontrolle von Diagnosen oder Therapievorschlägen von der KI. Auch ist das notwendige Maß an Zusatzinformationen aktuell nicht vorhersagbar. Das steht dem Einsatz von Sprachmodellen in der Lehre nicht im Wege, aber schmälert evtl. den Nutzen gegenüber der Arbeit eines menschlichen Dozenten, der einen Fachtext erstellt. Im klinischen Einsatz hingegen erscheint die Möglichkeit von zusätzlichen Informationen für die Frage häufiger gegeben. Dabei ist es eine wichtige Information für Behandler, welche Befunde noch fehlen, um eine Diagnose zu stützen. Der hohe Anteil an abgelehnten Fragen wegen fehlender Befunde zeigt, dass die aktuelle Version des Sprachmodells bei der Detektion solcher Situationen bereits weit fortgeschritten ist.

Insbesondere beim Einsatz in der Arbeit von Assistenzärzten können Sprachmodelle mit geeigneter Lernumgebung in Zukunft eine wichtige Unterstützung für den klinischen Alltag geben [18, 19]. Dabei ist entscheidend, dass die Lernumgebungen der Sprachmodelle optimiert werden, also auf eine adäquate Präsentation aller Patientengruppen geachtet und Quellen mit falschen Informationen aus den Lernumgebungen entfernt werden. Ebenso sollte man für die Anwendung durch deutsche Muttersprachler auch darauf achten, die Lernumgebung, wo möglich, zu einem größeren Anteil mit deutschsprachigen Quellen zu speisen. Die Priorisierung von Quellen wie medizinischen Leitlinien ist bisher nicht hinreichend berücksichtigt.

## Fazit

Als besonders geeignet scheint dabei der Einsatz von KI in den Rettungsstellen und Notaufnahmen, in der Intensiv- und Notfallmedizin, wo solche Sprachmodelle die Arbeit der Assistenzärzte durch Hinweise zur weiteren Diagnostik und zu Verdachtsdiagnosen unterstützen könnten. Für den Einsatz von KI-Sprachmodellen direkt am Patienten müssen jedoch noch einige Fähigkeiten von KI-Sprachmodellen optimiert werden. Ohne ärztliche Supervision, die im Zweifelsfall auch die Verantwortung für die Ergebnisse der KI übernehmen muss, scheint der Einsatz von Sprachmodellen zum aktuellen Zeitpunkt weiterhin risikobehaftet und nicht realisierbar. Der Einsatz in der Lehre ist ebenfalls eine Option, aber Fragen an die Sprachmodelle sollten so viele Informationen wie möglich enthalten und müssen ebenso wie die Antworten auf klinische Fragen supervidiert und ggf. revidiert werden.

Folglich sehen wir vier Anforderungen an Sprachmodelle bei ihrem Einsatz in Klinik und Lehre:

1. Transparenz bei den Quellen, die das Sprachmodell für die Beantwortung der Frage genutzt hat.
2. Selbstreflexion über Informationen, die für die Beantwortung der Frage durch das Sprachmodell aktuell noch benötigt werden.
3. Abgleich der gegebenen Antwort mit den Empfehlungen von Leitlinien und die ständige Kontrolle auf Halluzinationen.
4. Für den Einsatz in deutschsprachigen Bereichen sollte eine überwiegend deutschsprachige Lernumgebung für die Sprachmodelle genutzt werden, um Fehler durch Übersetzungen zu vermeiden.
